# Clinical usefulness of lipid ratios to identify men and women with metabolic syndrome: a cross-sectional study

**DOI:** 10.1186/1476-511X-13-159

**Published:** 2014-10-10

**Authors:** Danijela Gasevic, Jiri Frohlich, GB John Mancini, Scott A Lear

**Affiliations:** Department of Biomedical Physiology and Kinesiology, Simon Fraser University, 2600-515 West Hastings, Vancouver, British Columbia V6B 5K3 Canada; Department of Pathology and Laboratory Medicine, University of British Columbia, Vancouver, BC V6T 2B5 Canada; Division of Cardiology, Providence Health Care, Healthy Heart Program, 1081 Burrard Street, Vancouver, BC V6Z 1Y6 Canada; Department of Medicine, University of British Columbia, 2775 Laurel Street, Vancouver, British Columbia V5Z 1M9 Canada; Faculty of Health Sciences, Simon Fraser University, Burnaby, British Columbia V5A 1S6 Canada

**Keywords:** Metabolic syndrome, Lipid ratios, Triglyceride-to-high-density-lipoprotein-cholesterol, Low-density-lipoprotein-cholesterol-to-high-density-lipoprotein-cholesterol, Non-high-density-lipoprotein-cholesterol-to-high-density-lipoprotein-cholesterol, and total cholesterol-to-high-density-lipoprotein-cholesterol

## Abstract

**Background:**

Waist circumference, a metabolic syndrome (MetSy) criterion, is not routinely measured in clinical practice making early identification of individuals with MetSy challenging. It has been argued that ratios of commonly measured parameters such as lipids and lipoproteins may be an acceptable alternative for identifying individuals with MetSy. The objective of our study was to explore clinical utility of lipid ratios to identify men and women with MetSy; and to explore the association between lipid ratios and the number of MetSy components.

**Methods:**

Men and women (N = 797) of Aboriginal, Chinese, European, and South Asian origin (35–60 years), recruited across ranges of body mass index (BMI), with no diagnosed cardiovascular disease (CVD) or on medications to treat CVD risk factors were assessed for anthropometrics, family history of CVD, MetSy components (waist circumference, blood pressure, glucose, triglycerides (TG), high-density-lipoprotein-cholesterol (HDL-C)), low-density-lipoprotein-cholesterol (LDL-C), nonHDL-C, and health-related behaviours.

**Results:**

Mean levels of lipid ratios significantly increased with increasing number of MetSy components in men and women (p < 0.05). After adjustment for age, ethnicity, smoking, alcohol consumption, physical activity, family history of CVD and BMI, (and menopausal status in women), all lipid ratios were associated with the number of MetSy components in men and women (Poisson regression, p < 0.001). Compared to the rest of the lipid ratios (ROC curve analysis), TG/HDL-C was best able to discriminate between individuals with and without MetSy (AUC = 0.869 (95% CI: 0.830, 0.908) men; AUC = 0.872 (95% CI: 0.832, 0.912) women). The discriminatory power of TC/HDL-C and nonHDL-C/HDL-C to identify individuals with MetSY was the same (for both ratios, AUC = 0.793 (95% CI: 0.744, 0.842) men; 0.818 (95% CI: 0.772, 0.864) women). Additionally, LDL-C/HDL-C was a good marker for women (AUC = 0.759 (95% CI: 0.706, 0.812)), but not for men (AUC = 0.689 (95% CI: 0.631, 0.748)). Based on a multiethnic sample, we identified TG/HDL-C cut-off values of 1.62 in men and 1.18 in women that were best able to discriminate between men and women with and without MetSY.

**Conclusions:**

Our results indicate that TG/HDL-C is a superior marker to identify men and women with MetSy compared to TC/HDL-C, LDL-C/HDL-C, and nonHDL-C/HDL-C.

**Electronic supplementary material:**

The online version of this article (doi:10.1186/1476-511X-13-159) contains supplementary material, which is available to authorized users.

## Background

Metabolic syndrome is a cluster of metabolic abnormalities associated with type 2 diabetes [1, 2], cardiovascular morbidity and mortality, and all-cause mortality [3, 4]. It is alarming that the prevalence of metabolic syndrome is high and on the rise in both developed and developing countries [5, 6]. Early identification and treatment of individuals with metabolic syndrome is imperative to prevent debilitating consequences associated with its development. Regardless of the approach for diagnosing metabolic syndrome, central obesity, as measured by waist circumference, is one of the main criteria for diagnosing metabolic syndrome [7–10]. However, waist circumference is not routinely measured in primary care [11], which makes early identification of individuals with metabolic syndrome challenging.

It has been argued that ratios of commonly measured parameters such as lipids and lipoproteins may be an acceptable alternative for identifying individuals with metabolic syndrome [12–17]. Evidence shows that lipid ratios perform better than individual lipids in predicting cardiovascular risk [18–21]. Identifying a ratio to serve as a quick and simple tool for identifying individuals at increased cardiometabolic risk may decrease complexity of and increase efficiency in identifying and monitoring those at risk; especially as electronic medical records become more commonplace or if useful biochemical ratios were to be reported in routine laboratory test results. However, research on clinical usefulness of lipid ratios to identify individuals with metabolic syndrome is scarce and mostly limited to specific population groups such as Ghanian [17], Korean [14, 16], Spanish [12], and Turkish [13]. Furthermore, some of the available studies researched limited number of ratios [13, 17] or provided no cut-offs for lipid ratios to help guide physicians in identifying individuals at cardiometabolic risk [14, 15]. Therefore, the objective of this study was to explore the clinical usefulness of lipid ratios to identify men and women with metabolic syndrome. Lipid ratios researched include: total cholesterol-to-high-density-lipoprotein-cholesterol (TC/HDL-C), triglyceride-to-high-density-lipoprotein-cholesterol (TG/HDL-C), low-density-lipoprotein-cholesterol-to-high-density-lipoprotein-cholesterol (LDL-C/HDL-C), and non-high-density-lipoprotein-cholesterol-to-high-density-lipoprotein-cholesterol (nonHDL-C/HDL-C). In addition, given that the number of metabolic syndrome components has been shown to predict incident cardiovascular disease (CVD) and type 2 diabetes [22], we additionally explored the association of lipid ratios with the number of metabolic syndrome components among men and women coming from the ethnically diverse population.

## Results

Participants were equally represented across sexes and ethnicity. Compared to women, men had lower levels of HDL-C and significantly higher levels of total cholesterol, LDL-C, nonHDL-C, triglycerides, lipid ratios, glucose, blood pressure, and waist circumference; also, alcohol consumption was more prevalent among men than among women (Table 1). Similarly, the average number of metabolic syndrome components and prevalence of metabolic syndrome were higher in men than in women.

**Table 1:** **Distribution of risk factors in men and women**

	Men 380	Women 417	*p*value
Age (years)	46.8 ± 8.7	47.5 ± 8.9	0.241
Ethnicity			0.704
Aboriginal	81 (21.3%)	98 (23.5%)	
Chinese	100 (26.3%)	118 (28.3%)	
European	97 (25.5%)	99 (23.7%)	
South Asian	102 (26.8%)	102 (24.5%)	
Current smokers	44 (11.6%)	35 (8.4%)	0.133
Current consumers of alcohol	128 (33.7%)	98 (23.5%)	0.001
Physical activity (min/week)	226 (100, 447)	208 (95, 424)	0.269
Family history of cardiovascular disease	168 (44.2%)	187 (44.8%)	0.857
Waist circumference (cm)	92.6 ± 11.2	85.1 ± 12.2	<0.001
Body mass index (kg/m^2^)	27.6 ± 4.3	27.3 ± 5.3	0.498
TC (mmol/L)	5.25 ± 0.97	5.23 ± 1.03	0.776
HDL-C (mmol/L)	1.13 ± 0.29	1.43 ± 0.35	<0.001
LDL-C (mmol/L)	3.30 ± 0.85	3.17 ± 0.92	0.046
TC/HDL-C	4.91 ± 1.46	3.86 ± 1.23	<0.001
LDL/HDL-C	3.07 ± 1.01	2.35 ± 0.91	<0.001
nonHDL-C	4.12 ± 0.99	3.80 ± 1.05	<0.001
nonHDL-C/HDL-C	3.81 (2.93, 4.63)	2.68 (2.01, 3.45)	<0.001
TG	1.48 (0.99, 2.22)	1.17 (0.83, 1.63)	<0.001
TG/HDL-C	1.34 (0.82, 2.24)	0.86 (0.54, 1.22)	<0.001
Systolic BP (mmHg)	117 (110, 124)	115 (106, 126)	0.025
Diastolic BP (mmHg)	79.2 ± 9.7	75.5 ± 9.2	<0.001
Glucose (mmol/L)	5.30 (5.00, 5.60)	5.10 (4.80, 5.40)	<0.001
With MetSy	119 (31.3%)	104 (24.9%)	0.045
Number of MetSy components	1.84 ± 1.40	1.63 ± 1.22	0.026

Categorical variables presented as n (%), and sex differences were explored using Chi-square test. Continuous variables presented as mean ± SD if normally distributed or median (25%, 75%) if skewed. Sex differences explored using t-test and Mann U Whitney test for normally distributed and skewed data, respectively. TC – total cholesterol, HDL-C – high-density lipoprotein cholesterol, LDL-C – low-density lipoprotein cholesterol, TC/HDL-C – ratio of TC and HDL-C, TG – triglycerides, TG/HDL-C – ratio of TG and HDL-C, nonHDL-C/HDL – ratio of nonHDL-C and HDL-C, LDL-C/HDL-C – ratio of LDL-C and HDL-C, MetSy – metabolic syndrome.

**Figure 1:**
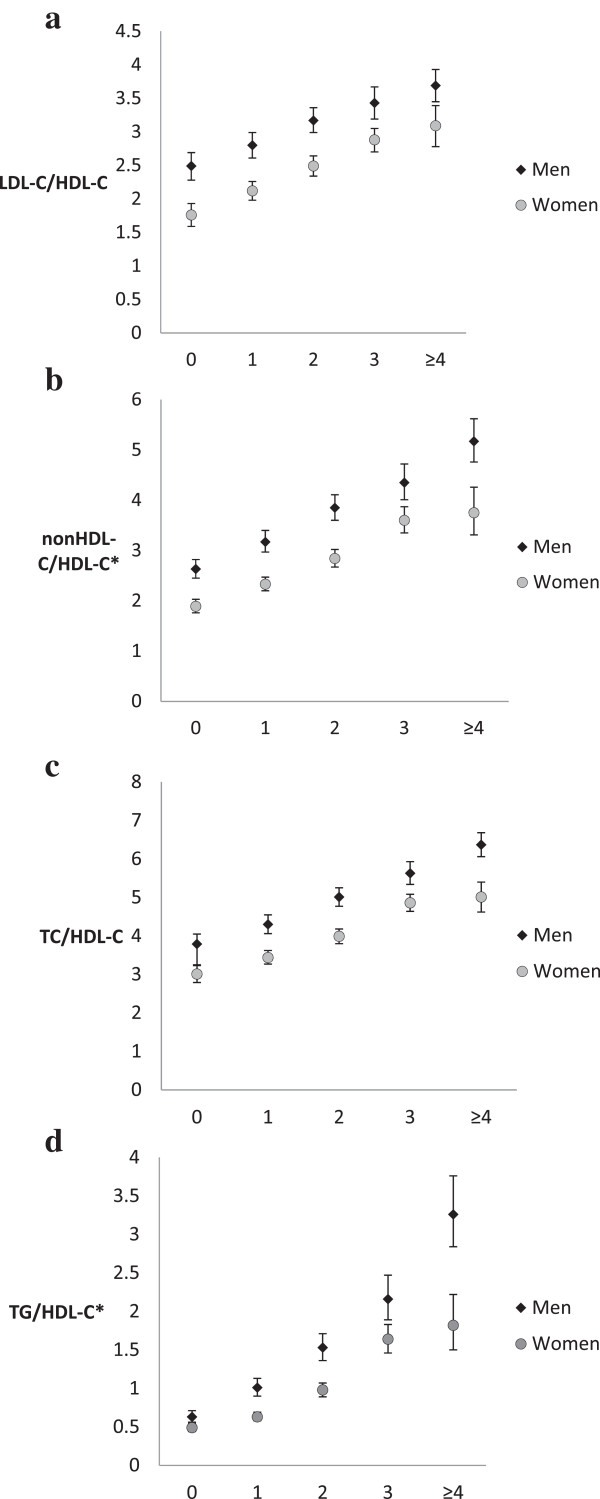
**Mean levels of lipid ratios across number of metabolic syndrome components in men and women (1A, 1B, 1C and 1D).** All means adjusted for age and ethnicity and presented as mean (95% CI). *geometric means. Bonferroni-corrected pairwise comparisons (0 vs. 1; 0 vs. 2; 0 vs. 3; 0 vs. ≥4; 1 vs. 2; 1 vs. 3; 1 vs. ≥4; 2 vs. 3; 2 vs. ≥4; and 3 vs. ≥4 ): *Men*: Except for the pair 3 vs. ≥4 for LDL-C/HDL-C, all other pairwise comparisons of means of TC/HDL-C, TG/HDL-C, LDL-C/HDL-C and nonHDL-C/HDL-C across the number of metabolic syndrome components were significant at p < 0.05. *Women*: Except for the pair 3 vs. ≥4, all other pairwise comparisons of means of TC/HDL-C, TG/HDL-C, LDL-C/HDL-C and nonHDL-C/HDL-C across the number of metabolic syndrome components were significant at p < 0.05. TG/HDL-C: triglyceride-to-high-density-lipoprotein-cholesterol, TC/HDL-C: total cholesterol-to-high-density-lipoprotein-cholesterol, LDL-C/HDL-C: low-density-lipoprotein-cholesterol-to-high-density-lipoprotein-cholesterol, nonHDL-C/HDL-C: non-high-density-lipoprotein-cholesterol-to-high-density-lipoprotein-cholesterol.

Mean levels of lipid ratios were on average higher in men than in women and significantly increased with increasing number of metabolic syndrome components in both men and women (Figure 1, Additional file 1: Table S1). In both men and women, mean levels of lipid ratios in individuals with zero metabolic syndrome components were significantly lower from their counterparts with 1 or more metabolic syndrome components (p < 0.05). In men and women, except for LDL-C/HDL-C ratio, there was a significant difference between mean levels of lipid ratios across number of metabolic syndrome components (p < 0.05). However, in both men and women, no significant difference was found between mean levels of LDL-C/HDL-C of participants with 3 and those with 4/5 metabolic syndrome components (p > 0.05). Furthermore, the results of partial correlation analyses (age and ethnicity adjusted) reveal that in both men and women, compared to the rest of the lipid ratios, TG/HDL-C showed stronger correlations with waist circumference, blood pressure and blood glucose (Additional file 2: Table S2). In addition, the results of Poisson regression analyses indicate that, after adjustment for age, ethnicity, smoking, alcohol consumption, physical activity, family history of CVD and body mass index (BMI) (and menopausal status in women), all lipid ratios were significantly associated with the number of metabolic syndrome components in both men and women (Table 2).

**Table 2:** **The association between lipid ratios and the number of metabolic syndrome components**

	Men	Women
	Exp (B (95% CI)	Exp (B) (95% CI)
TC/HDL-C	1.262 (1.197, 1.330)	1.278 (1.204, 1.355)
ln_TG/HDL-C	1.875 (1.680, 2.092)	1.797 (1.603, 2.016)
LDL-C/HDL-C	1.236 (1.147, 1.332)	1.358 (1.243, 1.483)
ln_nonHDL-C/HDL-C	2.861 (2.258, 3.626)	2.633 (2.109, 3.286)

All Poisson regression models adjusted for age, ethnicity, smoking, alcohol consumption, physical activity, family history of cardiovascular disease, and BMI. Models for women additionally adjusted for menopause status. All models significant at p < 0.001. TC/HDL-C – ratio of total cholesterol and high-density lipoprotein cholesterol, TG/HDL-C – ratio of triglycerides and high-density lipoprotein cholesterol, nonHDL-C/HDL – ratio of non-high-density lipoprotein cholesterol and high-density lipoprotein cholesterol, LDL-C/HDL-C – ratio of low-density lipoprotein cholesterol and high-density lipoprotein cholesterol.

**Figure 2:**
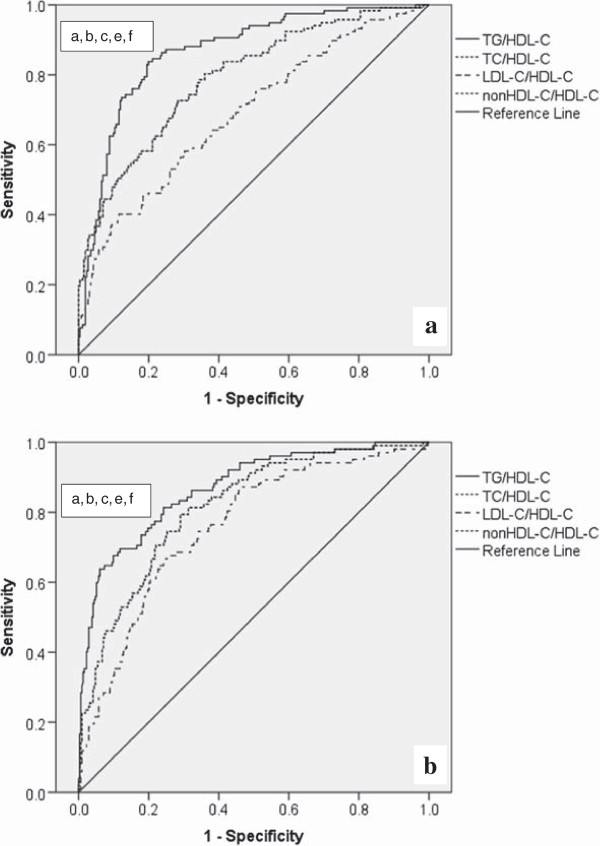
**Receiver operating characteristic curves for evaluating the usefulness of lipid ratios to identify men (A) and women (B) with metabolic syndrome.** The diagonal line indicates a test with an area under the receiver operating characteristic curve of 0.5. The areas under the curve for TC/HDL-C and nonHDL-C/HDL-C overlapped, as the discriminatory power of TC/HDL-C and nonHDL-C/HDL-C to identify individuals with metabolic syndrome was the same. The difference between areas under the curve (p ≤ 0.01): ^a^TG/HDL-C vs. TC/HDL-C. ^b^TG/HDL-C vs. nonHDL-C/HDL-C. ^c^TG/HDL-C vs. LDL-C/HDL-C. ^d^TC/HDL-C vs. nonHDL/HDL-C. ^e^TC./HDL-C vs. LDL-C/HDL-C. ^f^nonHDL-C/HDL-C vs. LDL-C/HDL-C. TG/HDL-C: triglyceride-to-high-density-lipoprotein-cholesterol, TC/HDL-C: total cholesterol-to-high-density-lipoprotein-cholesterol, LDL-C/HDL-C: low-density-lipoprotein-cholesterol-to-high-density-lipoprotein-cholesterol, nonHDL-C/HDL-C: non-high-density-lipoprotein-cholesterol-to-high-density-lipoprotein-cholesterol.

Using ROC curve analyses, we plotted sensitivity over 1-specificity for each of the ratios in both men and women (Figure 2). Compared to the rest of the lipid ratios, TG/HDL-C was best able to discriminate between apparently healthy men and women with and without metabolic syndrome (AUC = 0.869 (95% CI: 0.830, 0.908) for men; AUC = 0.872 (95% CI: 0.832, 0.912) for women). The discriminatory power of TC/HDL-C and nonHDL-C/HDL-C to identify individuals with metabolic syndrome was the same (for both ratios, AUC = 0.793 (95% CI: 0.744, 0.842) for men; 0.818 (95% CI: 0.772, 0.864) for women). In addition, based on Hosmer and Lemeshow’s criteria [23] LDL-C/HDL-C was an “acceptable” marker to discriminate between women with and without metabolic syndrome (AUC = 0.759 (95% CI: 0.706, 0.812)), however this was not the case for men (AUC = 0.689 (95% CI: 0.631, 0.748)). In both men and women, the analysis that tested the statistical significance of the differences between areas under the curve revealed significant difference between areas under the curves for all the pairs of lipid ratios (Figure 2). The exception is the pair of TC/HDL-C and nonHDL-C/HDL-C for which AUCs were the same. Lipid ratio cut-off values, with their respective sensitivities and specificities, for identifying individuals with metabolic syndrome are provided in Table 3.

**Table 3:** **Results of receiver-operating curve analyses featuring the thresholds of lipid ratios with their respective sensitivities and specificities for diagnosing metabolic syndrome in men and women**

	Men	Women
Lipid ratio	Criterion value (Sensitivity, specificity)	Criterion value (Sensitivity, specificity)
TG/HDL-C	1.62 (84.0%, 80.1%)	1.18 (70.2%, 88.2%)
TC/HDL-C	5.05 (73.1%, 71.6%)	3.91 (81.7%, 68.7%)
LDL-C/HDL-C*	3.79 (40.2%, 88.5%)	2.53 (67.6%, 74.8%)
nonHDL-C/HDL-C	4.05 (73.1%, 71.6%)	2.91 (79.8%, 70.9%)

*According to the AUC, LDL-C/HDL-C showed to be a good marker of metabolic syndrome in women but not in men.

## Discussion

The purpose of this study was to explore the clinical utility of lipid ratios to identify men and women with metabolic syndrome. Additionally we explored the association between lipid ratios and number of metabolic syndrome components. Our results indicate that increases in lipid ratios are significantly associated with increase in the number of metabolic syndrome components in both men and women after adjusting for age, ethnicity, smoking status, alcohol consumption, physical activity, family history of CVD, and BMI (and menopausal status in women). Compared to TC/HDL-C, LDL-C/HDL-C, and nonHDL-C/HDL-C, TG/HDL-C was shown to be a better clinical marker to discriminate between individuals with and without metabolic syndrome. These results were consistent for men and women.

The results of our study are in line with those based on Spanish, Korean, and Japanese populations where increases in lipid ratios were shown to be associated with increase in the number of metabolic syndrome components [12, 14, 15]. Our study extends the previous findings by reporting the positive association between lipid ratios and the number of metabolic syndrome components in a multiethnic population; this relationship persisted after adjusting for ethnicity and other factors known to be associated with metabolic syndrome.

The observed strong relationship between lipid ratios and the number of metabolic syndrome components may suggest lipid ratios as potential simple tools for early identification of individuals with constellation of cardiometabolic abnormalities. However, the actual lipid ratio cut-offs to guide clinicians in identifying individuals with metabolic syndrome are rarely reported. The studies available looked at a limited number of lipid ratios and pertained to specific groups such as Spanish population [12] or a population of Ghanian women [17]. We extend the available findings by reporting lipid ratio cut-off values for identifying men and women with metabolic syndrome. According to our results, in both men and women, TG/HDL-C was shown to be a better marker to identify individuals with metabolic syndrome compared to TC/HDL-C, LDL-C/HDL-C, and nonHDL-C/HDL-C. Based on a multiethnic sample, we identified TG/HDL-C cut-off values of 1.62 in men and 1.18 in women that were best able to discriminate between men and women with and without metabolic syndrome. These numbers were comparable but slightly lower than those reported by Cordero et al. [12] who clinically assessed a large working population of Spanish men and women and reported TG/HDL-C values of 2.75 and 1.65 as cut-offs for identifying men and women with metabolic syndrome, respectively. The observed differences in cut-offs reported in the above mentioned study and the ones reported in this study are most likely due to a difference in population groups studied.

It has recently been reported that TG/HDL-C predicts the development of CVD as effectively as the diagnosed metabolic syndrome [24]. Similarly, TG/HDL-C was found to predict coronary heart disease and CVD mortality as well as or even better than does metabolic syndrome in men [25]. However, the detection of an elevated TG/HDL-C is not meant to replace metabolic syndrome diagnosis in clinical practice; and it should rather be considered as a simple tool to quickly identify patients at increased cardiometabolic risk for whom further risk evaluation and clinical intervention are needed [25]. Indeed, it has recently been reported in the study in young adults that, compared to metabolic syndrome diagnostic, TG/HDL-C may be able to identify a greater number of individuals at risk; however a use of a metabolic syndrome diagnostics approach identifies individuals with an accentuated cardiometabolic risk profile [26]. While metabolic syndrome diagnosis may provide more comprehensive approach to identifying present cardiometabolic risk factors in individuals, this diagnostics is challenged by the fact that waist circumference, one of the integrative components of metabolic syndrome, is not commonly measured. Indeed, it has recently been reported that WC is routinely measured by only 6% of primary care physicians [11]. If translated, it may mean that more than 90% of primary care physicians would not be able to diagnose individuals with metabolic syndrome given that one of its components is not routinely measured. In contrast, both TG and HDL-C are routinely measured in clinical practice, and TG/HDL-C could be readily calculated (or provided by the laboratory) to serve as a quick tool to identify men and women at increased cardiometabolic risk. This approach to use TG/HDL-C to identify individuals at risk for whom further care is needed may reduce time and complexity for initial diagnosis of people with constellation of cardiometabolic abnormalities.

Other research groups have also proposed TG/HDL-C as a potential, simple tool to identify patients at increased risk for CVD. The evidence shows a strong association between TG/HDL-C and insulin resistance as measured by glucose clamp [27, 28], modified insulin suppression test [29], and homoeostasis model assessment index [29, 30]. This association was also found to be independent of waist circumference [31]. Further, others found TG/HDL-C to be an independent predictor of future type 2 diabetes mellitus [25, 32] and its related microvascular complications [33]; coronary heart disease [34, 35]; major cardiovascular events including overall death, myocardial infarction, and unstable angina that required revascularization [36] and those including angina pectoris, myocardial infarction, myocardial revascularization, and fatal or nonfatal stroke [37]; and first coronary event irrespective of BMI [38]. The strong relationship between TG/HDL-C and CVD may be founded in the atherogenic properties of TG/HDL-C. It has been reported that increase in TG/HDL-C is significantly associated with decrease in LDL particle size and increase in fractional esterification rates of cholesterol in plasma depleted of apoB-lipoproteins; hence the proposed name for TG/HDL-C being atherogenic index of plasma [39]. In light of available evidence, including the results of our study, it seems reasonable to conclude that TG/HDL-C may serve as a useful marker to identify individuals at increased risk of CVD.

Several limitations to our study should be considered. This study is a cross-sectional design, thus longitudinal studies are needed to explore whether the association between TG/HDL-C and metabolic syndrome persists or changes over time in men and women. Further, the M-CHAT participants were recruited across a range of BMI, so this study may not be representative of the general population. However, recruiting people across ranges of BMI allows for the opportunity to explore the associations between lipid ratios and metabolic syndrome in the population with a range of body sizes. Also, the results of our analyses indicate a significant association between lipid ratios and number of metabolic syndrome components independent of BMI. Moreover, our analyses were adjusted for age, ethnicity, smoking, status, alcohol consumption, physical activity, family history of CVD and BMI (and menopausal status in women) but not for diet known to influence plasma TG levels. Of importance, TG/HDL-C cut-points for identifying individuals at risk may vary across ethnicity and race [40, 41]. Also, TG/HDL-C was shown not to be a reliable risk marker in individuals of South Asian [41] and African American origin [42, 43]. While statistical power did not allow us to additionally stratify our analyses by ethnicity, given the established ethnic differences in the way body fat is accumulated [44, 45] and ethnic differences in cardiometabolic risk [46, 47], we believe that further research featuring ethnic-specific analyses is warranted.

## Conclusions

In conclusion, our study shows strong positive association between lipid ratios and metabolic syndrome in apparently healthy men and women (without CVD and not on CVD-related medications) drawn from an ethnically diverse population. TG/HDL-C appeared to be a superior marker compared to TC/HDL-C, LDL-C/HDL-C, and nonHDL-C/HDL-C, and it is useful for identifying both men and women with metabolic syndrome. Namely, TG/HDL-C readings of 1.62 or greater in men and 1.18 or greater in women can help primary care physicians easily identify individuals at increased risk for CVD. Early identification of individuals at risk would allow for an early implementation of lifestyle and medication strategies, while TG/HDL-C could be further used to evaluate the success of such strategies.

## Methods

Apparently healthy men and women were recruited as part of the Multi-Cultural Community Health Assessment Trial (M-CHAT) designed to compare body fat distribution of Aboriginal, Chinese, and South Asian populations to that of a European population and explore how fat distribution relates to CVD risk factors [48]. Details on recruitment of study participants have already been published [48]. In brief, eligible participants were apparently healthy individuals between ages of 30 and 65 of Aboriginal, Chinese, European and South Asian origin residing in the Vancouver mainland. To ensure a range of body fat mass across groups, participants were recruited from the following BMI (kg/m^2^) ranges: 18.5-24.9 (low range), 25.0-29.9 (middle range), and 30 and over (upper range). Given that it was challenging to identify Aboriginal men with a BMI of less than 25, Aboriginal men of any BMI were recruited. Similarly, due to a difficulty in identifying Chinese individuals with a BMI of 30 kg/m^2^ and higher, the target for the upper BMI range was changed to a BMI of 28 and higher. Individuals with a fluctuation in weight that was greater than 2.5 kg three months prior to the assessment date, and those with diagnosed CVD or on medications to treat CVD risk factors, were not eligible for the study. Ethnicity was self-reported. All study participants provided informed consent for the study, and the study was approved by the Simon Fraser University Research Ethics Board.

All participants were assessed for socio-demographics and risk factors. Following the standard protocols for blood sample collection, shipment, and processing, fasting blood samples were collected from each participant at the research site and assessed for lipids and glucose at St. Paul’s Hospital in Vancouver, Canada. Glucose, TC, TG, and HDL-C were determined using standard protocols by ADVIA 1650 analyzer (Bayer Health Care, LLC., New Jersey, USA). Friedwald formula was used to calculate LDL-C [49]. Height and weight were measured, and BMI was calculated as weight in kilograms divided by height in metres squared. Waist circumference represented an average of two measurements taken upon expiration at the mid point between the low rib margin and iliac crest. Blood pressure was recorded as mean of 5 measurements taken after 10 minutes seated rest in the left arm using BpTRU model BPM-200 oscillometric office blood pressure monitor (VSM MedTech Ltd., Coquitlam, British Columbia). Smoking status (current smokers/non-smokers), alcohol consumption (currently consume alcohol/do not consume alcohol) and family history of CVD (yes/no) were determined by self-report. Women self-reported their menopausal status by choosing from the following options (women were provided a definition for each of the options): pre-menopausal (regular menstrual cycles), peri-menopausal (menopausal transition with symptoms such as menstrual irregularity), post-menopausal (end of menstrual cycles, at least 12 months since the last menstrual period), or hysterectomy (surgical removal of the uterus). Menopausal status variable used as a confounder in regression analyses consisted of two categories, pre-menopausal (pre-menopausal and peri-menopausal) and menopausal (post-menopausal and hysterectomy). Physical activity was measured using the Modifiable Physical Activity questionnaire previously used in multi-ethnic populations and determined by self-report [50, 51]. A person would qualify as having metabolic syndrome if he/she had three out of 5 risk factors present (hypertension, hypertriglyceridaemia, lowered HDL-C, hyperglycaemia, and central obesity) using the most recent harmonized definition of the metabolic syndrome where ethnic specific thresholds for waist circumference were used [10].

### Statistical analyses

Only participants with complete data for all metabolic syndrome components were included to the study (N = 797). Considering significant sex differences in body fat distribution and cardiometablic risk [52–54], all statistical analyses were performed separately for men and women. Categorical variables were presented as counts and percentages, and differences between men and women were explored using Chi-square test. Continuous variables were presented as means ± standard deviation (SD) if normally distributed, or medians with 25^th^ and 75^th^ percentiles if not normally distributed. Differences between men and women in the distribution of continuous variables were explored using t-test and Mann U Whitney test for normally distributed and skewed data, respectively.

Age and ethnicity adjusted levels of lipid ratios across the number of metabolic syndrome components were determined using general linear modelling (Bonferroni-corrected *post hoc* comparisons). In our apparently healthy multiethnic study population, there was a limited number of participants with 4 (n = 66) and 5 (n = 18) metabolic syndrome components; therefore, we combined these two categories. Poisson regression analysis was used to explore the association between lipid ratios and the number of metabolic syndrome components. All models were adjusted for age, ethnicity, smoking, alcohol consumption, physical activity, family history of CVD and BMI. Models for women were additionally adjusted for menopausal status.

The clinical utility of lipid ratios to identify individuals with metabolic syndrome was explored using receiver-operating characteristic curve (ROC) analysis. Plots of sensitivity (true positives) versus 1 minus specificity (false positives) were constructed in both men and women for each lipid ratio. Area under the curve (AUC) was calculated to explore which lipid ratio showed highest accuracy in predicting metabolic syndrome. AUC is a measure of discrimination, and AUC of 0.5, 0.6 ≤ AUC < 7, 7 ≤ AUC < 0.8, 0.8 ≤ AUC < 0.9, and ≥ 0.9 corresponds to no discrimination, poor, acceptable, excellent, and outstanding discrimination, respectively [23]. Optimal cut-off point of each lipid ratio to identify individuals with metabolic syndrome corresponded to a maximum value of Youden index that was calculated as sensitivity + specificity – 1 [55, 56]. Additionally, the statistical significance of the difference between areas under the curves was tested using the methods of De Long et al. [57]. Analyses were performed using Statistical Package for Social Sciences (SPSS) version 19 except for the comparisons of ROC curves that were performed using MedCalc statistical software. P values of less than 0.05 were considered statistically significant.

## Electronic supplementary material

Additional file 1: Table S1: Mean levels (95% CI) of lipid ratios across increasing number of metabolic syndrome components. (DOCX 15 KB)

Additional file 2: Table S2: Partial correlations (age and ethnicity adjusted) between lipid ratios and metabolic syndrome components in men (A) and women (B). (DOCX 14 KB)

## Abbreviations

AUC: Area under the curve
BMI: Body mass index
CVD: Cardiovascular disease
LDL-C/HDL-C: Low-density-lipoprotein-cholesterol-to-high-density-lipoprotein-cholesterol
M-CHAT: Multi-Cultural Health Assessment Trial
nonHDL-C/HDL-C: non-high-density-lipoprotein-cholesterol-to-high-density-lipoprotein-cholesterol
ROC: Receiver-operating characteristic curve
SD: Standard deviation
SPSS: Statistical Package for Social Sciences
TC/HDL-C: Total cholesterol-to-high-density-lipoprotein-cholesterol
TG/HDL-C: Triglyceride-to-high-density-lipoprotein-cholesterol.
