# Supplementary material for: Clinical usefulness of lipid ratios to identify men and women with metabolic syndrome: a cross-sectional study
Source: Lipids Health Dis. 2014 Oct 10;13:159. doi: 10.1186/1476-511X-13-159 (PMC4210572; doi:10.1186/1476-511X-13-159)
Supplement: Supplementary file 1 — Additional file 1: Table S1: Mean levels (95% CI) of lipid ratios across increasing number of metabolic syndrome components. (DOCX 15 KB) [file 12944_2014_1141_MOESM1_ESM.docx]

Additional file 1: Table S1. Mean levels (95%CI) of lipid ratios across increasing number of metabolic syndrome components

|  | Men |  |  |  |  | Women |  |  |  |  |
| --- | --- | --- | --- | --- | --- | --- | --- | --- | --- | --- |
|  | 0 | 1 | 2 | 3 | 4/5 | 0 | 1 | 2 | 3 | 4/5 |
| TC/HDL-C | 3.79 (3.25, 4.05) | 4.30 (4.06, 4.55) | 5.01 (4.77, 5.25) | 5.63 (5.34, 5.93) | 6.37 (6.06, 6.68) | 3.01 (2.79, 3.23) | 3.44 (3.27, 3.62) | 3.99 (3.80, 4.18) | 4.86 (4.64, 5.08) | 5.01 (4.62, 5.40) |
| TG/HDL-C* | 0.63 (0.56, 0.71) | 1.01 (0.90, 1.13) | 1.53 (1.37, 1.71) | 2.16 (1.89, 2.47) | 3.26 (2.84, 3.76) | 0.49  (0.44, 0.55) | 0.63 (0.58, 0.69) | 0.98 (0.89, 1.07) | 1.64 (1.46, 1.83) | 1.82 (1.50, 2.22) |
| LDL-C/HDL-C | 2.49 (2.28, 2.69) | 2.80  (2.61, 2.99) | 3.17 (2.99, 3.36) | 3.43 (3.19, 3.67) | 3.69 (3.45, 3.93) | 1.76 (1.59, 1.93) | 2.12 (1.98, 2.26) | 2.49 (2.34, 2.64) | 2.88 (2.70, 3.05) | 3.09 (2.78, 3.39) |
| nonHDL-C/HDL-C* | 2.63 (2.45, 2.82) | 3.17 (2.97, 3.40) | 3.85 (3.60, 4.11) | 4.35 (4.01, 4.72) | 5.17 (4.76, 5.62) | 1.89 (1.76, 2.03) | 2.33 (2.20, 2.47) | 3.60 (3.35, 3.87) | 3.75 (3.31, 4.26) | 2.84 (2.67, 3.02) |

Results of general linear modelling; Means adjusted for age and ethnicity; *Geometric means (95% CI). Bonferroni-corrected pairwise comparisons (0 vs. 1; 0 vs. 2; 0 vs. 3; 0 vs. ≥4; 1 vs. 2; 1 vs. 3; 1 vs. ≥4; 2 vs. 3; 2 vs. ≥4; and 3 vs. ≥4 ): *Men*: Except for the pair 3 vs. ≥4 for LDL-C/HDL-C, all other pairwise comparisons of means of TC/HDL-C, TG/HDL-C, LDL-C/HDL-C and nonHDL-C/HDL-C across the number of metabolic syndrome components were significant at p<0.05. *Women*: Except for the pair 3 vs. ≥4, all other pairwise comparisons of means of TC/HDL-C, TG/HDL-C, LDL-C/HDL-C and nonHDL-C/HDL-C across the number of metabolic syndrome components were significant at p<0.05.
