# Supplementary material for: Clinical usefulness of lipid ratios to identify men and women with metabolic syndrome: a cross-sectional study
Source: Lipids Health Dis. 2014 Oct 10;13:159. doi: 10.1186/1476-511X-13-159 (PMC4210572; doi:10.1186/1476-511X-13-159)
Supplement: Supplementary file 2 — Additional file 2: Table S2: Partial correlations (age and ethnicity adjusted) between lipid ratios and metabolic syndrome components in men (A) and women (B). (DOCX 14 KB) [file 12944_2014_1141_MOESM2_ESM.docx]

Additional file 2: Table S2. Partial correlations (age and ethnicity adjusted) between lipid ratios and metabolic syndrome components in men (A) and women (B)

| A | WC | ln TG | HDL-C | ln SBP | DBP | ln Glu |
| --- | --- | --- | --- | --- | --- | --- |
| TC/HDL-C | 0.313 | 0.700 | -0.741 | 0.089^NS^ | 0.131* | 0.330 |
| ln TG/HDL-C | 0.351 | 0.958 | -0.745 | 0.096^NS^ | 0.136** | 0.350 |
| LDL-C/HDL-C | 0.233 | 0.404 | -0.637 | 0.070^NS^ | 0.110* | 0.226 |
| ln nonHDL-C/HDL-C | 0.306 | 0.700 | -0.784 | 0.086^NS^ | 0.137** | 0.317 |

*p<0.05, **p<0.01, NS – non significant; all others significant at p<0.001

| B | WC | ln TG | HDL-C | ln SBP | DBP | ln Glu |
| --- | --- | --- | --- | --- | --- | --- |
| TC/HDL-C | 0.240 | 0.643 | -0.704 | 0.116* | 0.147** | 0.194 |
| ln TG/HDL-C | 0.358 | 0.950 | -0.717 | 0.184 | 0.210 | 0.223 |
| LDL-C/HDL-C | 0.206 | 0.461 | -0.654 | 0.105* | 0.130** | 0.171 |
| ln nonHDL-C/HDL-C | 0.280 | 0.649 | -0.754 | 0.155** | 0.175 | 0.202 |

*p<0.05, **p<0.01, NS – non significant; all others significant at p<0.001

TC: total cholesterol, HDL-C: high-density lipoprotein cholesterol, TG: triglycerides, LDL-C: low-density lipoprotein cholesterol, WC: waist circumference, SBP: systolic blood pressure, DBP: diastolic blood pressure, Glu: blood glucose.
